# Development of a Clinical Prediction Model for Systemic Therapy Escalation in Oral Lichen Planus: A Two-Center Retrospective Cohort Study

**DOI:** 10.3390/medicina62102041

**Published:** 2026-10-21

**Authors:** Mihaela Paula Toader, Cristina Popa, Cristina Colac-Boțoc, Ana Maria Sciucă, Anastasia Vicovan, Daciana Elena Brănișteanu

**Affiliations:** 1 Department of Surgicals, Faculty of Dental Medicine, Grigore T. Popa University of Medicine and Pharmacy, 700115 Iasi, Romania; toaderpaula@gmail.com (M.P.T.); ana.filioreanu@umfiasi.ro (A.M.S.); 2 Dermatology Clinic, Railways University Hospital, 700506 Iasi, Romania; cristina.botoc28@gmail.com (C.C.-B.); anastasiavicovan@gmail.com (A.V.); debranisteanu@yahoo.com (D.E.B.); 3 Ambulatory of Oral and Maxillofacial Surgery of the “St. Spiridon” County Clinical Emergency Hospital, 700106 Iași, Romania; 4 Department of Dermatology, Faculty of Medicine, Grigore T. Popa University of Medicine and Pharmacy, 700115 Iasi, Romania

**Keywords:** oral lichen planus, systemic therapy, prediction model

## Abstract

*Background and Objectives:* Oral lichen planus (OLP) is a chronic immune-mediated disorder in which the need for systemic therapy remains difficult to predict using standardized clinical criteria. This retrospective two-center cohort study aimed to develop a multivariable clinical prediction model for systemic therapy escalation in patients with histopathologically confirmed OLP. *Material and Methods:* Medical records of 141 adult patients treated between 2022 and 2025 were reviewed. Baseline demographic, clinical, and laboratory variables were analyzed using univariate analyses, followed by multivariable logistic regression with backward stepwise selection and Firth penalized likelihood estimation. Discrimination was assessed by the area under the ROC curve (AUC), and internal validation was performed by bootstrap resampling (1000 samples) with repetition of the full selection procedure. *Results:* Four independent predictors of systemic therapy were identified: concomitant cutaneous lichen planus (penalized odds ratio (OR) 13.96, 95% CI 3.94–55.84), autoimmune thyroiditis (OR 12.67, 3.26–54.88), genital lichen planus (OR 10.21, 2.36–51.57) and oral Candida superinfection (OR 4.48, 1.10–18.82). The apparent AUC was 0.889 (95% CI 0.801–0.976); after bootstrap internal validation, the optimism-corrected AUC was 0.84 and the calibration slope 0.73, indicating moderate overfitting. Candida superinfection was the least stable predictor (selected in 43% of bootstrap samples). A prognostic risk matrix was developed to estimate the probability of systemic therapy escalation according to different combinations of predictors. *Conclusions:* The identified clinical factors and derived risk matrix may provide a preliminary framework for risk stratification and individualized therapeutic decision-making in patients with OLP. However, given the retrospective design and limited number of outcome events, these findings should be interpreted cautiously. Prospective external validation in larger independent cohorts is required before the model can be considered for routine clinical use.

## 1. Introduction

Oral lichen planus (OLP) is a chronic immune-mediated inflammatory disorder affecting the oral mucosa, characterized by T-cell-mediated destruction of basal keratinocytes and persistent interface inflammation. It affects approximately 1–2% of the adult population and occurs predominantly in middle-aged and older women. OLP is recognized by the World Health Organization (WHO) as an oral potentially malignant disorder because of its established, although relatively low, risk of malignant transformation into oral squamous cell carcinoma [1,2].

Clinically, OLP presents as reticular, plaque-like, atrophic, erosive, or bullous lesions, with erosive and atrophic forms being the most symptomatic and associated with substantial impairment of oral health-related quality of life [3,4].

The diagnosis of OLP is established through a combination of characteristic clinical features and histopathological confirmation according to the modified WHO and American Academy of Oral and Maxillofacial Pathology (AAOMP) criteria. Although most patients present with lesions confined to the oral cavity, a subset also exhibits cutaneous or genital involvement, reflecting a more generalized autoimmune process. Because OLP follows a chronic relapsing-remitting course, affected patients require long-term clinical surveillance to control disease activity and to detect early malignant transformation [5,6].

Topical corticosteroids remain the first-line treatment and are effective for most patients with localized disease [7,8]. Alternative topical therapies, including calcineurin inhibitors, may be used in refractory cases. However, patients presenting with extensive erosive disease, multisite involvement, or persistent symptoms despite optimized topical therapy frequently require systemic immunomodulatory treatment. The decision to escalate therapy remains largely empirical and is generally based on symptom severity and clinician experience rather than on standardized, evidence-based criteria. Consequently, identifying patients who are likely to require systemic treatment at an early stage remains an important unmet clinical need [7,8].

Several demographic, clinical, and biological factors have been associated with severe OLP, including erosive disease, extraoral involvement, autoimmune comorbidities, and systemic inflammatory markers. Nevertheless, these factors have mostly been studied individually, and no validated multivariable model is currently available to estimate the probability of systemic therapy at the time of diagnosis. The absence of such predictive tools contributes to considerable variability in clinical decision-making and may delay appropriate treatment in patients with aggressive disease [9,10].

The originality of the present study lies in shifting the focus from the conventional assessment of factors associated with OLP severity or treatment response toward a prediction-based approach specifically addressing systemic therapy escalation. While previous studies have primarily examined individual clinical features, comorbidities, or inflammatory markers in relation to disease severity and therapeutic outcomes, their combined predictive value for subsequent systemic treatment escalation remains insufficiently explored. By integrating routinely available baseline clinical variables into a multivariable model and translating the resulting estimates into an individualized risk matrix, the present study provides a clinically oriented framework for early risk stratification that may complement conventional severity-based therapeutic assessment.

The present two-center retrospective cohort study was designed to address this gap by identifying independent clinical and biological predictors associated with the need for systemic therapy in patients with histopathologically confirmed OLP. Using multivariable logistic regression, we developed a prediction model aimed at facilitating early risk stratification and supporting individualized therapeutic decision-making.

## 2. Materials and Methods

This retrospective two-center cohort study was conducted to identify clinical and biological factors associated with systemic therapy escalation in patients with OLP. Medical records of adult patients with histopathologically confirmed OLP who were evaluated between January 2022 and December 2025 at the Dermatology Clinic of the Railways University Hospital and the Ambulatory of Oral and Maxillofacial Surgery of the “St. Spiridon” County Clinical Emergency Hospital, Iași, Romania, were reviewed. These institutions represent two major referral centers involved in the diagnosis and management of OLP in Iași, providing complementary dermatological and oral and maxillofacial care to patients from the same geographic region. Both centers contributed patients during the same study period, using the same predefined patient-identification strategy and eligibility criteria, as well as comparable diagnostic, follow-up, and therapeutic protocols. A total of 141 patients fulfilled the eligibility criteria and were included in the final analysis.

The study is reported in accordance with Strengthening the Reporting of Observational Studies in Epidemiology (STROBE) recommendations.

Patients were eligible for inclusion if they were ≥18 years of age and had a definitive diagnosis of OLP established according to the modified WHO 2003 diagnostic criteria and the AAOMP 2016 recommendations [1].

Patients were excluded if they had histopathological evidence of severe epithelial dysplasia, carcinoma in situ, or oral squamous cell carcinoma at the initial evaluation. Additional exclusion criteria included oral lichenoid contact reactions associated with dental restorative materials, drug-induced oral lichenoid lesions, graft-versus-host disease, and other autoimmune or vesiculobullous disorders capable of mimicking OLP clinically or histopathologically.

Demographic characteristics, including age and sex, were retrieved from the medical records. Clinical data comprised disease duration, clinical phenotype, anatomical distribution of oral lesions, extraoral involvement, systemic comorbidities, current medications, and treatment history.

Clinical OLP phenotypes were recorded as erosive, atrophic, reticular, plaque-like, or other morphological patterns. As multiple clinical patterns could coexist in the same patient, phenotype categories were not mutually exclusive. Lesion distribution was recorded according to the involved oral sites, including the buccal mucosa, tongue, gingiva, palate, and labial mucosa. Extraoral manifestations, including cutaneous and genital lichen planus, were systematically assessed and documented, with the diagnosis confirmed by histopathological examination.

Disease duration was calculated from the date of diagnosis to the Initial evaluation included in the study and categorized as recent (<1 year), intermediate (1–3 years), or long-standing (>3 years).

Information regarding systemic comorbidities was collected from the medical records, including hypertension (HTN), type 2 diabetes mellitus (T2DM), dyslipidemia, obesity, chronic heart failure, autoimmune thyroiditis, other thyroid disorders, psychiatric disorders, hepatitis B virus (HBV) and hepatitis C virus (HCV) infection anemia, and other autoimmune diseases. Autoimmune thyroiditis was recorded based on a pre-existing documented diagnosis or following thyroid screening and subsequent endocrinological confirmation. The presence of dental restorative materials and oral Candida superinfection (diagnosed based on compatible clinical findings and direct microscopic examination of lingual scrapings) was also recorded.

Laboratory parameters included the erythrocyte sedimentation rate (ESR) and the neutrophil-to-lymphocyte ratio (NLR), calculated from routine complete blood count analyses performed during the initial clinical assessment.

The primary outcome was systemic therapy escalation during baseline and during the six-month follow-up period.

Therapeutic management was categorized as no active topical or systemic treatment, topical treatment alone, systemic treatment alone, or combined topical and systemic treatment.

Treatment strategy was individualized according to disease extent, severity, and clinical course. Systemic therapy was considered for patients with extensive or severe erosive OLP, multisite LP involvement, and/or insufficient response to topical therapy, generally reassessed after approximately three months of treatment with tacrolimus and/or clobetasol. Systemic agents included corticosteroids, azathioprine, methotrexate, and dapsone.

Treatment response, when available from follow-up records, was categorized as absent, partial, or complete clinical response.

Statistical analyses were performed using IBM SPSS Statistics version 31.0 (IBM Corp., Armonk, NY, USA) and Python 3.11. Continuous variables were assessed for normality using the Shapiro–Wilk test. Normally distributed variables are presented as mean ± standard deviation (SD), whereas non-normally distributed variables are reported as median and interquartile range (IQR). Categorical variables are expressed as absolute frequencies and percentages. Baseline characteristics associated with systemic therapy escalation were initially evaluated using univariate analyses. Categorical variables were compared using Pearson’s chi-square test or Fisher’s exact test, as appropriate; for contingency tables larger than 2 × 2 with expected frequencies <5, the Fisher–Freeman–Halton exact test was used. Crude odds ratios (ORs) with 95% confidence intervals (CIs) were calculated for binary variables. Continuous variables were compared using the independent samples *t*-test or the Mann–Whitney U test according to data distribution. Variables associated with systemic therapy escalation in the univariate analyses (*p* < 0.05) were considered as candidate predictors and were entered into a multivariable binary logistic regression model and selected by backward stepwise elimination based on the likelihood-ratio test (removal at *p* > 0.10). The adequacy of the sample size was assessed according to the criteria proposed by Riley et al. for the development of prediction models with a binary outcome, based on the observed outcome prevalence and model R^2^; because of the limited number of outcome events, the final model was re-estimated using Firth’s penalized likelihood logistic regression. Model discrimination was assessed by the area under the receiver operating characteristic curve (AUC), with 95% confidence intervals calculated using DeLong’s method, and overall performance by the Nagelkerke R^2^ and the Brier score. Calibration was assessed by the calibration slope and calibration-in-the-large. Internal validation was performed using bootstrap resampling (1000 samples drawn with replacement). Overall accuracy was summarized by the Brier score and the scaled Brier score. As a sensitivity analysis, a LASSO-penalized logistic regression including all candidate predictors (standardized) was fitted, with the penalty parameter chosen by 20-times repeated 5-fold cross-validation. Predicted probabilities were calculated for each of the 16 possible predictor profiles, with 95% confidence intervals obtained by non-parametric bootstrap of the penalized model (2000 resamples, percentile method); observed proportions in each profile are reported with 95% Wilson confidence intervals. All statistical tests were two-sided, with the level of statistical significance set at α = 0.05; accordingly, *p*-values < 0.05 were considered statistically significant. Detailed descriptive statistics, complete univariate analysis, and supplementary regression output are provided in the Supplementary Materials.

The study was conducted in accordance with the principles of the Declaration of Helsinki and was approved by the Institutional Ethics Committee (Approval No. 12449/2026). Owing to the retrospective nature of the study, informed consent from all participants was waived by the Ethics Committee according to institutional regulations.

## 3. Results

### 3.1. Baseline Characteristics

A total of 141 patients with histopathologically confirmed OLP were included, comprising 101 women (71.6%) and 40 men (28.4%) (Table S1). The mean age of the overall cohort was 65.38 ± 13.20 years (median: 67.00 years; range: 21–95 years). The mean age was 65.53 ± 13.31 years in women and 65.00 ± 13.09 years in men, with no statistically significant difference between the two groups (*p* = 0.873). The distribution across age groups was also similar in women and men (<50 years: 7.9% vs. 10.0%; 50–64 years: 31.7% vs. 30.0%; 65–74 years: 38.6% vs. 35.0%; ≥75 years: 21.8% vs. 25.0%; *p* = 0.94), as was disease duration (median 4.0 vs. 6.0 years; *p* = 0.223) (Table S2). Regarding patient distribution across the two participating centers, 78 patients (55.3%) were included from the Oral and Maxillofacial Surgery center and 63 (44.7%) from the Dermatology center. Systemic therapy occurred in 5/78 patients (6.4%) and 18/63 patients (28.6%), respectively (*p* < 0.001) (Table S24).

Regarding therapeutic management, 24.1% of patients (*n* = 34/141) received neither topical nor systemic treatment, 59.6% (*n* = 84/141) received topical treatment alone, 3.5% (n = 5/141) received systemic treatment without concomitant topical therapy at, and 12.8% (*n* = 18/141) received combined topical and systemic treatment. Among the 23 patients included in the systemic-treatment group, systemic therapy was initiated at baseline evaluation in 5 patients (21.7%), while 18 patients (78.3%) initiated systemic treatment during the six-month follow-up.

The reticular subtype was the most common clinical presentation, occurring in 59.6% (*n* = 84/141) of patients, followed by the erosive subtype in 43.3% (*n* = 61/141), plaque-like lesions in 14.2% (*n* = 20/141), the atrophic subtype in 13.5% (n = 19/141), and other clinical variants in 8.5% (*n* = 12/141) (Table S3).

The most frequently involved oral sites were the buccal mucosa (*n* = 117/141) and tongue (*n* = 55/141), followed by the labial mucosa (*n* = 18/141), gingiva (*n* = 16/141), and hard palate (*n* = 10/141) (Table S4).

Extraoral LP involvement was documented at baseline, including cutaneous LP in 14.9% (*n* = 21/141), genital LP in 9.2% (*n* = 13/141), and involvement of other extraoral sites in 2.8% (*n* = 4/141) of patients (Table S4).

Most patients had a disease duration of more than 3 years (62.4%, *n* = 88/141), whereas intermediate (1–3 years) and recent (<1 year) disease durations were observed in 19.9% (*n* = 28/141) and 17.7% (*n* = 25/141), respectively. The mean disease duration was 6.71 ± 6.29 years (median: 5.00 years); disease duration did not differ significantly between women and men (median 4.0 vs. 6.0 years; Mann–Whitney U = 1754.0, *p* = 0.223) (Table S5).

At baseline, 61.7% of patients (*n* = 87/141) were receiving systemic medication for chronic comorbidities, whereas 12.1% (*n* = 17/141) reported the use of vitamin or trace element supplements. Metallic or restorative dental materials were documented in 67.4% of the study population (*n* = 95/141) (Table S6).

The most frequently documented materials were metal–composite restorations (*n* = 44), Gaudent alloy restorations (*n* = 14), metal–ceramic restorations (*n* = 12), metal–acrylic restorations (*n* = 10), and removable acrylic prostheses (*n* = 9). Less frequently recorded materials or prosthetic restorations included skeletal prostheses, ceramic or composite restorations, non-veneered metal restorations, amalgam, gold, and implant-supported prostheses. As some patients presented with more than one type of dental material or restoration, these categories were not mutually exclusive.

Comorbidities identified across the cohort included HTN (*n* = 81/141), T2DM (*n* = 33/141), dyslipidemia (*n* = 27/141), obesity (*n* = 20/141), chronic heart failure (*n* = 12/141), and psychiatric disorders (*n* = 11/141). Autoimmune thyroiditis and other associated thyroid pathologies were documented in 11.3% (*n* = 16/141) of patients. Positive HBV/HCV status was documented in 16.3% (*n* = 23/141) of patients, while anemia was present in 7.8% (*n* = 11/141). Oral Candida superinfection was documented in 12.1% (*n* = 17/141) of cases. Among patients with available laboratory data, the mean baseline ESR was 14.93 mm/h (±12.586 SD; median: 10.00), and the mean NLR was 2.18 ± 1.333 (median: 1.83) (Tables S7 and S9).

Among the 107 patients who received active topical and/or systemic treatment, treatment response was documented in 87 (81.3%): complete clinical response in 64 (73.6%), partial response in 14 (16.1%) and no clinical response in 9 (10.3%) (Table S8).

The evaluation of continuous parameters across the cohort demonstrated a total mean disease duration of 6.71 years (±6.288 SD), with a median duration of 5.00 years (IQR: 2.00 ÷ 11.25 years) and a maximum tracking span of 42.00 years (*n* = 141) (Table 1 and Table S9).

### 3.2. Univariate Analysis

To identify factors associated with the need for systemic treatment, baseline demographic, clinical, and biological characteristics were compared between patients managed without systemic therapy (*n* = 118) and those requiring systemic treatment (*n* = 23). The complete results of the univariate analyses are presented in Table 2 (Table S21). 

Gender was not associated with systemic therapy escalation (*p* = 0.202), while patients requiring systemic therapy were significantly younger than those managed with topical therapies (median age: 61 vs. 68 years, *p* = 0.035) and had a significantly shorter disease duration (median: 1 vs. 6 years, *p* = 0.013). Furthermore, recent-onset disease was significantly more frequent among patients requiring systemic therapy (*p* = 0.002) (Tables S10, S11, S15 and S16).

Among the clinical characteristics evaluated, the plaque-like phenotype was significantly associated with systemic therapy escalation (OR = 3.53, 95% CI: 1.23–10.19; *p* = 0.023) (Table S12). Extraoral involvement demonstrated the strongest univariate associations, with concomitant cutaneous lichen planus increasing the odds of requiring systemic treatment more than 13-fold (OR = 13.21, 95% CI: 4.56–38.28; *p* < 0.001), while genital lichen planus was associated with an approximately 18-fold increase in the odds of requiring systemic treatement (OR = 18.32, 95% CI: 4.98–67.36; *p* < 0.001). The atrophic phenotype was not observed among patients requiring systemic therapy and therefore showed an inverse association (*p* = 0.043) (Tables S13 and S14).

Among the evaluated comorbidities, autoimmune thyroiditis showed the strongest association with systemic treatment (OR = 7.33, 95% CI: 2.40–22.45; *p* < 0.001). Dyslipidemia (OR = 3.57, *p* = 0.017), obesity (OR = 3.53, *p* = 0.023), and oral Candida superinfection (OR = 3.43, *p* = 0.035) were also significantly associated with systemic treatment. In contrast, the presence of dental restorative materials was associated with a significantly lower likelihood of requiring systemic therapy (OR = 0.24, 95% CI: 0.09–0.61; *p* = 0.002) (Table S17).

Regarding laboratory parameters, neither baseline NLR (median 2.14 vs. 1.71; *p* = 0.163) nor ESR (*p* = 0.574) differed significantly between the two groups. Patient sex, lesion location, hypertension, diabetes mellitus, chronic heart failure, viral hepatitis, psychiatric disorders, anemia, and most of the remaining clinical variables showed no statistically significant association with systemic treatment (Table S18).

Overall, the univariate analysis identified younger age, shorter disease duration, plaque-like OLP, concomitant cutaneous and genital lichen planus, autoimmune thyroiditis, dyslipidemia, obesity and oral Candida superinfection as candidate predictors for multivariable modelling.

### 3.3. Multivariable Logistic Regression and Risk Stratification

Variables that demonstrated statistically significant associations in the univariate analyses were entered into a multivariable binary logistic regression model using a backward stepwise selection procedure to identify independent predictors of systemic therapy escalation. Variables resulting in complete separation were excluded from the multivariable model to ensure model stability. Accordingly, the atrophic OLP phenotype was not included because no patients with this clinical presentation required systemic therapy. In addition, treatment response was excluded from the regression analysis because it represents a therapeutic outcome rather than a baseline predictive variable.

The final multivariable model retained four independent predictors significantly associated with systemic therapy (Table 3). After penalized estimation, concomitant cutaneous LP showed the strongest association with systemic therapy escalation (penalized OR = 13.96, 95% CI: 3.94–55.84; *p* < 0.001), followed by autoimmune thyroiditis (OR = 12.67, 95% CI: 3.26–54.88; *p* < 0.001), genital LP (OR = 10.21, 95% CI: 2.36–51.57; *p* = 0.002) and oral Candida superinfection (OR = 4.48, 95% CI: 1.10–18.82; *p* = 0.037). The same four predictors were selected in the complete-case analysis including NLR, and NLR was not retain.

Among the retained variables, cutaneous LP and autoimmune thyroiditis showed the highest adjusted effect sizes, whereas genital involvement and oral Candida superinfection also contributed independently to the final predictive model. The persistence of these variables after multivariable adjustment indicates that their association with the need for systemic treatment was independent of the remaining clinical and biological characteristics included in the analysis.

**Figure 1:**
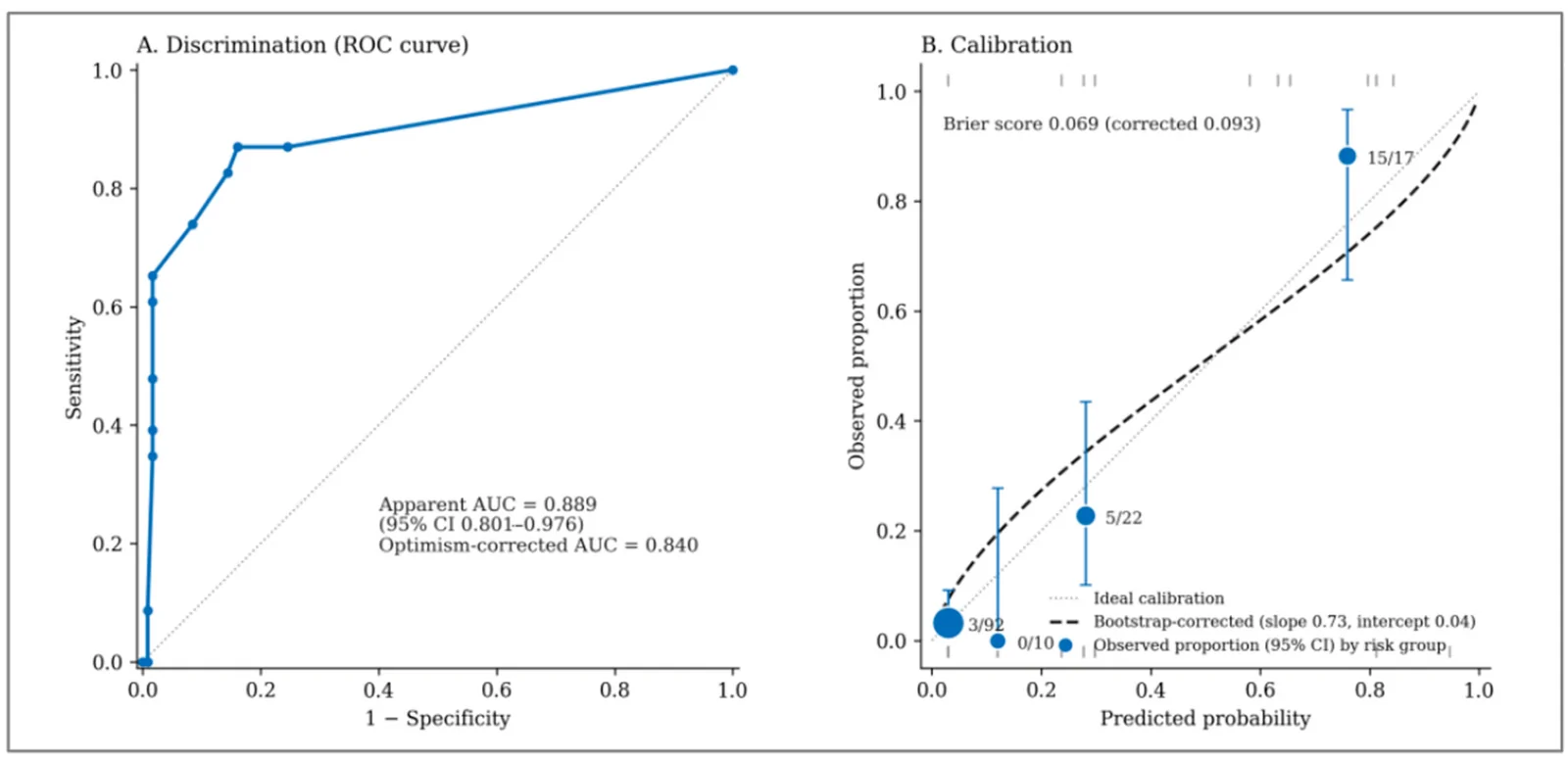
Performance of the final prediction model (Firth penalized logistic regression with intercept correction; *n* = 141, 23 events). (**A**) Receiver operating characteristic curve, with apparent AUC (95% CI, DeLong) and optimism-corrected AUC after bootstrap internal validation. (**B**) Calibration plot: observed proportion of patients with systemic therapy escalation (95% Wilson CI) against mean predicted probability in four predicted-risk groups (<5%, 5–20%, 20–50%, ≥50%; numbers indicate events/patients, marker size proportional to group size); the dotted line represents ideal calibration and the dashed line the bootstrap-corrected calibration line (intercept 0.04, slope 0.73). Tick marks show the distribution of predicted probabilities in patients with (top) and without (bottom) systemic therapy escalation.

The performance of the final model is summarized in Table 4 and Figure 1. The model showed an apparent AUC of 0.889 (95% CI: 0.801–0.976) and a Nagelkerke R^2^ of 0.539. After bootstrap internal validation of the complete model-development procedure (backward selection followed by penalized estimation), the optimism-corrected AUC was 0.840 and the calibration slope 0.73, indicating moderate overfitting; the corresponding values for the unpenalized (maximum-likelihood) procedure were 0.838 and 0.69 (Table S21).

Mean predicted and observed risks agreed closely in the development data (expected/observed events: 23.0/23). In the calibration plot (Figure 1B), observed proportions were 3/92 (3.3%) in patients with a predicted probability <5%, 0/10 in the 5–20% group, 5/22 (22.7%) in the 20–50% group and 15/17 (88.2%) in patients with a predicted probability ≥50%, with wide confidence intervals in the intermediate groups. The optimism-corrected calibration intercept and slope were 0.04 and 0.73, respectively (0.69 for the unpenalized procedure), indicating moderate overfitting. The Brier score was 0.069 (optimism-corrected 0.093), compared with 0.137 for a non-informative model, corresponding to scaled Brier scores of 0.50 and 0.32; the Nagelkerke R^2^ was 0.539 (optimism-corrected 0.357). At a predicted-probability threshold of 20%, the model identified 39 patients as high risk, with a sensitivity of 87.0%, a specificity of 83.9%, a positive predictive value of 51.3% and a negative predictive value of 97.1% (apparent estimates; Table S23).

Autoimmune thyroiditis, cutaneous LP and genital LP were selected in 96.1%, 92.0% and 85.2% of bootstrap samples, respectively, whereas oral Candida superinfection was selected in 43.2%, and the remaining candidates in 17.9–43.4% (Table S22). In the LASSO sensitivity analysis, the same four predictors had the largest coefficients.

After adjustment for centre, cutaneous LP (OR 11.82, 95% CI 3.33–46.89), genital LP (OR 8.67, 95% CI 2.02–43.15) and autoimmune thyroiditis (OR 10.26, 95% CI 2.50–45.99) remained strongly associated with systemic therapy escalation, whereas the association with oral Candida superinfection was attenuated (OR 3.91, 95% CI 0.94–16.57; *p* = 0.060); centre itself was not independently associated with the outcome (OR 2.05, 95% CI 0.59–7.62; *p* = 0.257), and no significant centre-by-predictor interaction was found. The model predicted 6.9 and 16.1 events in the two centres (observed 5 and 18, respectively) (Table S24).

### 3.4. Development of the Prognostic Risk Matrix and Model Performance

**Figure 2:**
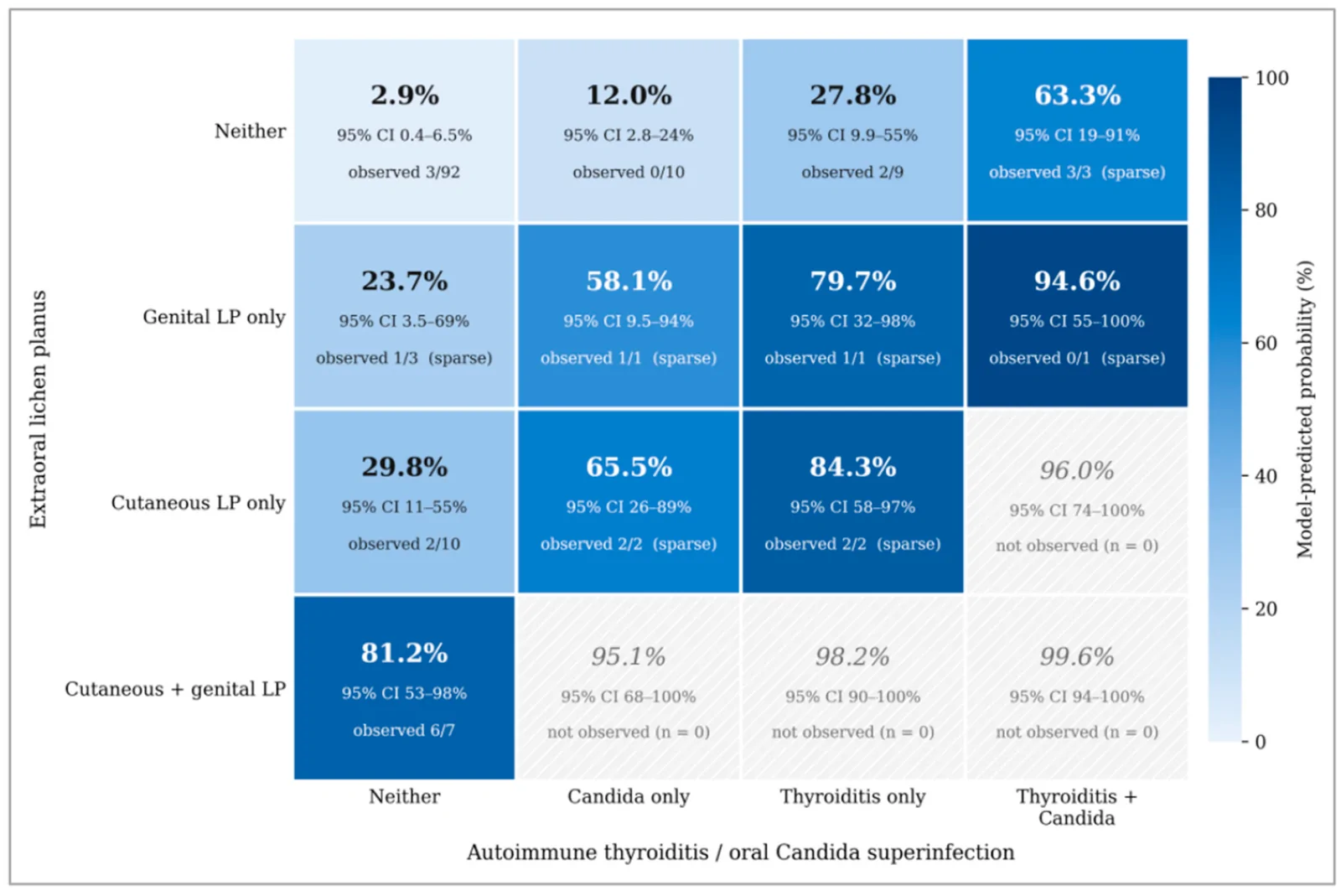
Predicted probability of systemic therapy escalation according to combinations of the four independent predictors retained in the multivariable model.

The regression coefficients derived from the final multivariable logistic regression model were subsequently used to estimate the individual probability of systemic therapy escalation according to all possible combinations of the four independent predictors retained in the final model. Based on these coefficients, predicted probabilities were calculated for each of the sixteen possible clinical profiles, resulting in the development of the prognostic risk matrix presented in Figure 2. This matrix translates the statistical model into an individualized probability estimate, providing a practical representation of the cumulative effect of the independent predictors identified in the multivariable analysis.

The estimated probability of requiring systemic therapy increased with the number and combination of predictors present (Figure 2, Table S21). Patients without any predictor, who represented the majority of the cohort (92/141, 65.2%), had an estimated probability of 2.9% (observed 3/92). The presence of a single predictor increased the estimated probability to 12.0–29.8%, and two-predictor profiles corresponded to estimated probabilities between 58.1% and 84.3%; the combination of cutaneous and genital LP, observed in seven patients, was associated with an estimated probability of 81.2% (observed 6/7). However, the matrix should be interpreted in light of the distribution of patients across profiles: four of the 16 possible profiles, including the profile with all four predictors, were not observed, and several others included fewer than five patients. Probabilities corresponding to these profiles are model extrapolations and are identified as such in Figure 2. Confidence intervals were wide for sparsely represented profiles (e.g., genital LP, thyroiditis with Candida superinfection); in the case of unobserved profiles they reflect only the statistical uncertainty of the additive model, not the uncertainty of extrapolating beyond the observed data (Table S20).

The coexistence of two independent predictors resulted in a marked increase in the estimated probability of systemic therapy, with predicted values ranging between 56.9% and 88.6%. More specifically, the estimated probability was 56.93% for genital LP combined with oral Candida superinfection, 68.24% for autoimmune thyroiditis combined with oral Candida superinfection, 73.77% for cutaneous LP combined with oral *Candida* superinfection, 78.45% for genital LP combined with autoimmune thyroiditis, 82.65% for concomitant cutaneous and genital LP, and 88.56% for cutaneous LP combined with autoimmune thyroiditis. Thus, among two-predictor profiles, the highest estimated probabilities were observed for combinations involving cutaneous LP, particularly when associated with autoimmune thyroiditis. When three predictors were simultaneously present, the estimated probability consistently exceeded 95% across all evaluated clinical profiles. The theoretical profile including all four independent predictors yielded the highest model-estimated probability (99.8%). However, several predictor combinations were sparsely represented or were not observed in the study cohort. Therefore, the number of patients actually observed for each of the 16 possible combinations is also reported in Figure 2, and probabilities corresponding to rare or unobserved profiles should be interpreted cautiously as model-derived estimates.

Collectively, these findings demonstrate a progressive increase in the estimated probability of systemic therapy escalation as additional independent predictors accumulated, ilustrating the ability of the model to stratify patients across a broad spectrum of predicted clinical risk.

From a clinical perspective, this matrix may help identify patients with a higher predicted probability of systemic therapy escalation, who could benefit from closer monitoring and earlier reassessment of treatment response. However, it should be regarded as a risk-stratification aid rather than a direct indication for initiating systemic therapy.

## 4. Discussion

### 4.1. Main Findings

OLP remains one of the most challenging chronic inflammatory disorders encountered in oral medicine because of its highly heterogeneous clinical presentation and unpredictable therapeutic course. Although topical corticosteroids remain the first-line treatment for most patients, the decision to escalate to systemic therapy is still largely based on clinical judgment, symptom severity, and individual physician experience, as standardized predictive criteria are currently lacking. Consequently, identifying patients who are unlikely to respond adequately to topical treatment remains a major unmet clinical need.

In the present two-center retrospective cohort study, we developed a multivariable prediction model to identify baseline clinical characteristics associated with the need for systemic therapy in patients with OLP. Four variables remained independently associated with systemic treatment after multivariable adjustment: concomitant cutaneous LP, autoimmune thyroiditis, genital LP, and oral Candida superinfection. These predictors were subsequently incorporated into a prognostic risk matrix capable of estimating the individual probability of systemic therapy escalation using routinely available clinical information. After correction for optimism, the model showed good discrimination (AUC 0.84), but the calibration slope of 0.73 indicated moderate overfitting, so that its predicted probabilities should be regarded as preliminary until confirmed in external data.

An notable finding of the present study is that the strongest predictors of systemic therapy were not the extent of oral lesions alone, but rather variables reflecting broader disease involvement and systemic immune dysregulation. Extraoral manifestations, autoimmune comorbidity, and alterations of the oral microenvironment remained independently associated with systemic treatment, suggesting that patients requiring systemic therapy may represent a distinct clinical subset characterized by more complex immune-mediated disease. These findings extend previous observations regarding disease severity in OLP and provide a framework for objective baseline risk stratification [11,12]. Although previous studies have identified clinical factors associated with OLP severity, symptom burden, and treatment outcome, evidence regarding the factors associated with systemic treatment in OLP remains limitied [13,14]. Rather than relying exclusively on subjective clinical assessment or symptom severity, our model integrates four readily obtainable clinical variables into an individualized probability estimate. Although external validation is required before routine implementation, this approach provides a framework for a more standardized and evidence-based strategy for therapeutic decision-making in patients with OLP.

### 4.2. Extraoral Disease Extension and Autoimmune Burden

The present study identified extraoral lichen planus involvement as the strongest determinant of systemic therapy escalation. After multivariable adjustment, both cutaneous and genital LP remained independent predictors, while autoimmune thyroiditis also demonstrated a strong association with the need for systemic treatment. These findings suggest that the requirement for systemic therapy is influenced not only by the severity of oral lesions but also by the overall extent of immune-mediated disease. Patients presenting with multisite mucocutaneous involvement appear to represent a distinct clinical phenotype characterized by more extensive immune dysregulation and a greater likelihood of inadequate response to topical therapy.

Our findings are consistent with previous studies reporting that patients with concomitant cutaneous or genital involvement generally exhibit more extensive disease and experience a greater therapeutic burden than those with isolated oral lesions [15,16]. Although extraoral manifestations have traditionally been regarded as indicators of disease extension, our results suggest that they may also serve as clinically relevant predictors of therapeutic escalation. From a practical perspective, the presence of cutaneous or genital lesions during the initial evaluation should therefore prompt a more comprehensive assessment of disease severity and closer therapeutic monitoring, particularly in patients presenting with symptomatic erosive disease.

The strong association observed between autoimmune thyroiditis and systemic therapy further supports the concept that OLP should be considered within the broader spectrum of immune-mediated disorders rather than as an isolated oral condition. Previous systematic reviews and meta-analyses have consistently demonstrated a higher prevalence of autoimmune thyroid disease among patients with OLP, suggesting the existence of shared immunological mechanisms [17,18,19]. In particular, molecular mimicry, shared genetic susceptibility, and dysregulated T-cell–mediated immune responses have been proposed as potential mechanisms linking these conditions. Experimental studies have also explored the expression of thyroid-related molecules within OLP lesions; however, the available evidence remains inconsistent, indicating that the biological mechanisms underlying the association between OLP and autoimmune thyroid disease have not yet been fully elucidated [20,21].

Although the present study was not designed to investigate pathogenic mechanisms, the observed associations are biologically plausible. OLP is characterized by a chronic T-cell-mediated immune response directed against basal keratinocytes, resulting in persistent epithelial injury and chronic mucosal inflammation [22]. The coexistence of oral, cutaneous, and genital lesions may therefore reflect a broader autoimmune process involving multiple epithelial sites rather than independent manifestations of disease. Such widespread immune activation may partly explain why these patients are less likely to achieve adequate disease control with localized topical therapy alone and more frequently require systemic immunomodulatory treatment.

Taken together, these findings suggest that extraoral disease involvement and autoimmune comorbidity may represent markers of overall disease burden rather merely accompanying clinical features. Their inclusion in the final multivariable model highlights their potential role in the early identification of patients at increased risk of systemic therapy escalation and supports their incorporation into baseline clinical assessment.

### 4.3. Oral Candida Superinfection and the Local Inflammatory Microenvironment

Oral Candida superinfection remained an independent predictor of systemic therapy escalation after multivariable adjustment, highlighting the potential contribution of local inflammatory factors to treatment outcomes in OLP. Although Candida colonization is frequently regarded as a secondary complication of chronic mucosal disease or prolonged corticosteroid therapy, our findings suggest that its presence at baseline may also identify patients with a higher probability of requiring systemic treatment. This observation suggests that local microbial alterations may contribute to disease persistence rather than being a simple consequence of topical corticosteroid use.

The relationship between OLP and oral candidiasis has been extensively investigated, although the reported prevalence varies considerably depending on the studied population, clinical phenotype, and microbiological methods [23,24,25]. In a retrospective study including 203 patients with OLP, Parlatescu et al. reported oral Candida colonization in approximately one-third of patients, with Candida albicans representing the predominant species. Although fungal colonization was not significantly associated with specific clinical phenotypes, the authors observed that superinfection was associated with increased symptom severity, including burning sensation, soreness, and mucosal discomfort, emphasizing its potential impact on disease burden [26].

Several mechanisms may explain the association between Candida superinfection and the need for systemic therapy. Chronic inflammatory lesions, particularly erosive and ulcerative forms of OLP, compromise epithelial barrier integrity and facilitate opportunistic fungal colonization. Conversely, persistent fungal colonization may further amplify local inflammation through continuous activation of innate and adaptive immune pathways, creating a self-perpetuating inflammatory microenvironment. This bidirectional interaction may reduce the effectiveness of topical therapies and contribute to persistent symptomatic disease requiring escalation to systemic treatment [23,24,25].

In addition to microbial dysbiosis, oxidative stress has emerged as an important contributor to OLP pathogenesis. Previous studies have demonstrated increased production of reactive oxygen species (ROS) and enhanced lipid peroxidation within active OLP lesions, promoting activation of inflammatory signaling pathways such as nuclear factor kappa (NF-κB) and the subsequent production of pro-inflammatory cytokines, including tumor necrosis factor alpha (TNF-α) and matrix metalloproteinases [15,27]. These mechanisms contribute to sustained epithelial injury and delayed mucosal healing. Although oxidative stress markers were not evaluated in the present study, they provide a biologically plausible explanation for the association observed between chronic inflammatory activity, fungal colonization, and therapeutic refractoriness.

From a clinical perspective, the identification of oral Candida superinfection as an independent predictor of systemic therapy escalation highlights the importance of careful baseline oral examination and appropriate management of fungal infection before and during OLP treatment. Whether early recognition and treatment of candidiasis may improve therapeutic outcomes or reduce the need for systemic therapy remains uncertain and warrants prospective investigation.

### 4.4. Clinical Implications

Although only four variables remained independently associated with systemic therapy escalation in the final multivariable model, several additional clinical and biological characteristics demonstrated significant associations in the univariate analyses. Younger age, shorter disease duration, recent-onset disease, obesity, dyslipidemia and plaque-like OLP were all more frequently observed among patients who ultimately required systemic treatment. Although these variables did not retain independent significance after multivariable adjustment, they provide valuable insights into the clinical profile of patients presenting with more severe disease.

A notable finding was the association between shorter disease duration and the subsequent need for systemic therapy. Patients requiring treatment escalation had a significantly shorter disease duration and were generally younger at presentation than those successfully managed with topical therapy alone. Rather than suggesting that OLP invariably follows a rapidly progressive course, these findings may indicate that a subset of patients presents with a more active disease phenotype characterized by earlier therapeutic failure. Similar observations have been reported by Wongpakorn et al., who identified older age and longer treatment duration as factors associated with delayed clinical remission following topical corticosteroid therapy [28]. These findings suggest that the clinical course of OLP is heterogeneous and that early disease activity may differ substantially between individual patients.

Metabolic comorbidities also demonstrated significant associations in the univariate analysis. Both obesity and dyslipidemia were more common among patients requiring systemic therapy, supporting previous evidence linking metabolic disorders with chronic low-grade systemic inflammation. Adipose tissue is recognized as an active endocrine organ capable of producing pro-inflammatory mediators, including TNF-α, interleukin-6 (IL-6), and interleukin-1β (IL-1β), which may contribute to sustained immune activation [15]. Although these variables were not retained in the final multivariable model, they may still influence disease severity indirectly through their interaction with other components of the inflammatory response.

Although NLR has been proposed as a simple marker of systemic inflammatory activity in several chronic inflammatory disorders, including OLP [29,30,31,32], baseline NLR did not differ significantly between patients with and without systemic therapy escalation in our cohort, and it did not improve the prediction model when added after multiple imputation of missing values. However, NLR was unavailable for 17% of patients, and larger prospective studies with systematic laboratory assessment are needed to clarify its potential prognostic value.

Taken together, these observations indicate that systemic therapy escalation in OLP is influenced by a complex interaction between clinical manifestations, autoimmune comorbidity, local inflammatory factors, and the patient’s systemic inflammatory profile. Although not all variables contributed independently to the final prediction model, they help define the broader clinical context in which treatment escalation occurs and may represent valuable candidates for future prospective studies incorporating larger patient cohorts and molecular biomarkers.

The principal clinical contribution of the present study lies in the development of a practical prediction model that may support the initial assessment of patients with OLP. Current therapeutic recommendations generally advocate topical corticosteroids as first-line therapy, whereas escalation to systemic treatment is primarily guided by clinical severity, symptom burden, and response to treatment. However, objective tools capable of estimating the probability of subsequent systemic therapy escalation based on baseline characteristics remain limited. The model proposed in this study represents an attempt to complement clinical judgment by providing an individualized estimate of the likelihood of treatment escalation based on routinely available clinical variables.

A key advantage of the proposed model is its simplicity. All four independent predictors retained in the final regression model, cutaneous LP, genital LP, autoimmune thyroiditis, and oral Candida superinfection, can be identified during the routine baseline evaluation without the need for specialized laboratory investigations or advanced imaging techniques. Consequently, the prediction model may be readily integrated into everyday clinical practice without increasing diagnostic complexity or healthcare costs.

Rather than functioning as a substitute for clinical judgment, the proposed risk matrix should be considered a decision-support tool. Patients presenting with a low estimated probability of systemic therapy escalation may be suitable candidates for conventional topical management and routine follow-up. On the other hand, individuals with progressively higher predicted probabilities may benefit from closer monitoring, earlier reassessment of treatment response, and multidisciplinary evaluation involving oral medicine specialists, dermatologists, and other relevant healthcare professionals when appropriate. Importantly, the model is intended to assist clinicians in identifying patients who may require closer surveillance rather than to prescribe a specific therapeutic strategy.

The progressive increase in estimated probabilities observed with the accumulation of independent predictors suggests that systemic therapy requirements are unlikely to depend on a single clinical characteristic alone. Instead, treatment escalation appears to reflect the combined effect of multiple clinical and biological factors, supporting a more individualized approach to patient assessment. Such an approach may help reduce delays in recognizing patients who are less likely to achieve adequate disease control with topical therapy alone while avoiding unnecessary systemic treatment in individuals with a lower predicted probability of escalation.

The combination-based analysis further illustrates the potential clinical value of considering predictors jointly rather than in isolation. Among two-predictor profiles, estimated probabilities of systemic therapy escalation ranged from 56.93% to 88.56%, with combinations involving cutaneous LP generally associated with higher predicted probabilities. Notably, the coexistence of cutaneous LP and autoimmune thyroiditis yielded the highest estimated probability (88.56%). These findings suggest that patients presenting with multiple baseline risk factors may represent a subgroup warranting closer clinical surveillance and earlier reassessment of therapeutic response.

Future validation should ideally be performed in a prospective, multicenter cohort including patients with newly diagnosed or actively managed OLP across different clinical settings. The four predictors identified in the present model, cutaneous LP, genital LP, autoimmune thyroiditis, and oral Candida superinfection, should be systematically assessed at baseline, together with additional potentially relevant variables such as disease severity, quality of life, psychosocial factors, smoking, and alcohol consumption. The primary outcome should remain the initiation of systemic therapy, assessed from baseline though a predefined follow-up period, while longer-term treatment escalation, treatment response, and disease progression may be considered secondary outcomes. Following successful external validation and appropriate calibration, the model could be translated into a simple point-based or digital risk calculator integrated into routine clinical assessment. Such a tool could support risk-based follow-up intensity and earlier therapeutic reassessment, while complementing rather than replacing clinical judgment.

### 4.5. Strengths and Limitations

The present study has several strengths. First, all patients fulfilled clinical and histopathological diagnostic criteria for oral lichen planus, minimizing the risk of diagnostic misclassification. Second, the study was conducted across two referral centers, increasing the heterogeneity of the study population and potentially improving the generalizability of the findings within similar clinical settings. Third, the use of multivariable logistic regression allowed the identification of independent predictors of systemic therapy escalation while accounting for potential confounding variables. Finally, the development of an individualized prognostic risk matrix represents an important step toward a more objective and standardized approach to therapeutic risk stratification in patients with OLP.

The presence of concomitant cutaneous or genital LP may have influenced the decision to initiate systemic therapy in patients with multisite involvement, introducing a potential risk of confounding by indication.

Several limitations should be acknowledged. First, the retrospective design inherently carries a risk of selection and information bias. The analysis relied on data documented in existing medical records, and the completeness and consistency of clinical information may therefore have varied between patients and between the two participating centers. Variables that were not routinely or systematically recorded could not be reliably assessed, potentially resulting in incomplete characterization of relevant clinical factors. Furthermore, the inclusion of patients evaluated at two referral centers may have introduced selection bias toward individuals with more complex or symptomatic disease, thereby limiting the generalizability of the findings to broader OLP populations. To reduce potential misclassification, we applied predefined eligibility criteria and included only patients with OLP established according to standardized clinical and histopathological diagnostic criteria. Nevertheless, residual information and selection bias cannot be excluded and should be considered when interpreting the associations and predictive performance observed in this cohort.

Second, the number of outcome events was limited (23 events). According to the criteria of Riley et al., the sample size was adequate for a model with four predictor parameters, but not for data-driven selection among 11 candidate parameters, which would have required approximately 250 patients and 41 events. Although penalized estimation and bootstrap validation were used to quantify and reduce overfitting, the optimism-corrected calibration slope (approximately 0.7) indicates that the predicted probabilities, particularly those at the extremes of the risk matrix, are likely to be overestimated in new patients. Moreover, oral Candida superinfection was selected in fewer than half of the bootstrap samples, and its contribution to the model should be regarded as uncertain. External validation in larger independent cohorts, with recalibration if necessary, is therefore required before the model can be applied in clinical practice.

The two participating centres differed in case mix and in the frequency of systemic therapy escalation, reflecting different referral pathways. Although the difference between centres was largely explained by the predictors included in the model and centre was not independently associated with the outcome, the small number of events per centre did not allow centre-specific models or cross-centre validation, and differences in treatment thresholds between centres cannot be completely excluded.

The predefined six-month follow-up period represents an additional limitation. Although this interval provided a consistent observation window across the cohort, some patients who did not initiate systemic therapy at baseline or during follow-up may have required systemic treatment after this period, potentially resulting in outcome misclassification. Therefore, the model should be interpreted as estimating the probability of systemic therapy initiation at baseline or during the subsequent six-month follow-up period, rather than the long-term need for systemic treatment. Longer prospective follow-up is warranted to assess systemic treatment initiation over time.

Another important limitation is the absence of external validation. Although the model demonstrated good discrimination after internal validation, its performance should be confirmed in independent patient populations before routine clinical implementation. Prospective multicenter studies involving larger cohorts will be essential to evaluate the reproducibility and generalizability of the proposed prediction model.

In addition, the retrospective design limited the range of candidate predictors that could be evaluated. Potentially relevant factors, including psychosocial variables, validated oral health-related quality-of-life measures, smoking status, and alcohol consumption, were not consistently documented in the medical records and therefore could not be incorporated into the analysis. These factors may influence symptom burden, disease perception, inflammatory status, and therapeutic decision-making and may provide additional predictive information beyond the variables included in the present model. Consequently, the four predictors retained in the final model should not be interpreted as an exhaustive representation of all factors potentially associated with systemic therapy escalation. Future prospective studies should incorporate standardized assessment of psychosocial factors, quality of life, and behavioral exposures to determine whether they improve the predictive performance of the model.

The present study also focused primarily on routinely available clinical variables. Several potentially relevant biological markers, including cytokine profiles, oxidative stress markers, and salivary biomarkers, were not available because of the retrospective study design. Future studies integrating clinical predictors with molecular biomarkers may further improve the predictive accuracy of individualized risk assessment.

Despite these limitations, the proposed model demonstrated good internally validated discrimination and provides an initial framework for objective risk stratification in patients with oral lichen planus. The identification of four readily obtainable clinical predictors may facilitate future refinement of prediction models and support the development of more individualized therapeutic strategies.

## 5. Conclusions

This study identified four independent baseline predictors of systemic therapy escalation in OLP: concomitant cutaneous LP, autoimmune thyroiditis, genital LP, and oral Candida superinfection. The combination of these predictors enabled the estimation of individualized probabilities of treatment escalation, with progressively higher predicted risk as multiple factors coexisted.

The resulting prognostic risk matrix provides a simple framework for early risk stratification using routinely available clinical variables and may help identify patients who could benefit from closer monitoring and earlier therapeutic reassessment. However, given the retrospective design and limited number of outcome events, the model should currently be considered a preliminary decision-support framework. Prospective external validation in larger, independent multicenter cohorts is required before its implementation in routine clinical practice.

## Figures and Tables

**Table 1:** Comprehensive baseline demographic, clinical, and biological profiles of the study cohort (*n* = 141).

Baseline Parameter	Distribution (No./Total No. of Patients in Cathegory or Mean ± SD)	Frequency (%) or Median (IQR/Range)
**Demographic profiles**		
Gender: Female	101/141	71.6%
Gender: Male	40/141	28.4%
Total Age (Years)	65.38 ± 13.201	Median: 67.00 (Range: 21–95)
Age: Female subgroup	65.53 ± 13.307	Median: 67.00 (IQR: 57.00 ± 74.00)
Age: Male subgroup	65.00 ± 13.089	Median: 67.00 (IQR:58.50 ± 74.75)
**Therapeutic regimens**		
Topical OLP treatment	102/141	72.3%
Systemic OLP therapy required	23/141	16.3%
Morphological OLP subtypes *		
Reticular subtype	84/141	59.6%
Erosive subtype	61/141	43.3%
Plaque-like subtype	20/141	14.2%
Atrophic subtype	19/141	13.5%
Other subtypes	12/141	8.5%
Anatomical subsites involved *		
Buccal mucosa	117/141	83.0%
Tongue	55/141	39.0%
Labial mucosa	18/141	12.8%
Gingival tissue	16/141	11.3%
Hard palate	10/141	7.1%
**Extra-oral manifestations**		
Cutaneous Lichen Planus	21/141	14.9%
Genital Lichen Planus	13/141	9.2%
Alternative Extra-oral Sites	4/141	2.8%
**Disease duration**		
Recent (<1 year)	25/141	17.7%
Intermediate (1–3 years)	28/141	19.9%
Long-standing (>3 years)	88/141	62.4%
Total Disease Duration (years)	6.71 ± 6.288	Median: 5.00 (Max: 42.00)
**Systemic background & comorbidities**		
Concurrent systemic medication	87/141	61.7%
Restorative dental materials present	95/141	67.4%
Hypertension	81/141	57.4%
Type 2 Diabetes Mellitus	33/141	23.4%
Dyslipidemia	27/141	19.1%
Obesity	20/141	14.2%
Autoimmune thyroiditis	16/141	11.3%
Other thyroid disorders	16/141	11.3%
Positive viral status (HCV/HBV)	23/141	16.3%
Oral Candida superinfection	17/141	12.1%
Mineral/Vitamin supplementation	17/141	12.1%
Chronic Heart Failure (ICC)	12/141	8.5%
Other Autoimmune Conditions **	12/141	8.5%
Psychiatric disorders	11/141	7.8%
Anemia	11/141	7.8%
Inflammatory Bowel Disease (IBD)	0/141	0.0%
**Continuous biological parameters**		
ESR	14.93 ± 12.586	Median: 10.00 (IQR: 5.00 ± 20.00; N = 70)
NLR	2.18 ± 1.333	Median: 1.83 (IQR: 1.40 ± 2.60; N = 117)
Categorical treatment response ***		
Complete clinical response	64/87	73.6%
Absent clinical response	9/87	10.3%
Partial clinical response	14/87	16.1%

* Note: Phenotype and subsite percentages do not sum to 100% due to overlapping concurrent configurations in individual patients. ** Includes Sjögren’s syndrome, celiac disease, lupus erythematosus, psoriasis, and pernicious anemia. *** Assessed in patients receiving active treatment (*n* = 107); response documented in 87.

**Table 2:** Univariate comparisons of clinical and biological factors stratified by OLP systemic therapy requirement.

Explored Parameter	No Systemic Therapy Required (n/118 or N)	Systemic Therapy Required (n/23 or N)	Statistical Test Value	Odds Ratio (OR) (95% CI)	Statistical *p*-Value
**Gender (Female)**	82/118 (69.5%)	19/23 (82.6%)	Pearson χ2 = 1.630	2.09 (0.66–6.57)	*p* = 0.202
**Age at Evaluation (Median, IQR)**	68.00 (60.00 ÷ 74.25)	61.00 (53.00 ÷ 69.00)	Mann-Whitney U = 980.000	—	*p* = 0.035 *
**Disease Duration (Median, IQR)**	6.00 (2.00 ÷ 12.00)	1.00 (0.25 ÷ 8.00)	Mann-Whitney U = 912.500	—	*p* = 0.013 *
**Disease Duration < 1 year**	15/118 (12.7%)	10/23 (43.5%)	Fisher’s exact	5.28 (1.97–14.16)	*p* = 0.001 **
**Plaque-like OLP Form**	13/118 (11.0%)	7/23 (30.4%)	Fisher’s exact	3.534 (1.226 ÷ 10.187)	*p* = 0.023 *
**Atrophic OLP Form**	19/118 (16.1%)	0/23 (0.0%)	Fisher’s exact	Not estimable (0 events)	*p* = 0.043 *
**Associated Cutaneous LP**	9/118 (7.6%)	12/23 (52.2%)	Fisher’s exact	13.212 (4.561 ÷ 38.276)	*p* < 0.001 **
**Associated Genital LP**	4/118 (3.4%)	9/23 (39.1%)	Fisher’s exact	18.321 (4.983 ÷ 67.363)	*p* ≤ 0.001 **
**Restorative Dental Materials**	86/118 (72.9%)	9/23 (39.1%)	Pearson χ2 = 9.975	0.239 (0.094 ÷ 0.607)	*p* = 0.002 **
**Dyslipidemia Comorbidity**	18/118 (15.3%)	9/23 (39.1%)	Fisher’s exact	3.571 (1.345 ÷ 9.480)	*p* = 0.017 *
**Obesity Comorbidity**	13/118 (11.0%)	7/23 (30.4%)	Fisher’s exact	3.534 (1.226 ÷ 10.187)	*p* = 0.023 *
**Autoimmune Thyroiditis**	8/118 (6.8%)	8/23 (34.8%)	Fisher’s exact	7.333 (2.396 ÷ 22.445)	*p* < 0.001 **
**Oral** ***Candida*** **superinfection**	11/118 (9.3%)	6/23 (26.1%)	Fisher’s exact	3.433 (1.122 ÷ 10.508)	*p* = 0.035 *
**Peripheral NLR (Median, IQR)**	1.71 (1.40 ÷ 2.60)	2.14 (1.67 ÷ 2.62)	Mann-Whitney U = 777.500	—	*p* = 0.163
**Partial Response to Treatment †**	5/69 (7.2%)	9/18 (50.0%)	Fisher-Freeman-Halton exact	—	*p* < 0.001 **

* *p* < 0.05; ** *p* < 0.01. Mann–Whitney U test (asymptotic, corrected for ties). † Among patients receiving active treatment with documented response (69 topical only, 18 systemic).

**Table 3:** Multivariable logistic regression model for systemic therapy escalation (*n* = 141; 23 events): maximum-likelihood and Firth penalized estimates.

Predictor	B (ML)	OR (ML)	95% CI (Wald)	B (Firth)	Penalized OR	95% CI (Profile)	*p* (Penalized LR)
Constant	−3.665	—	—	−3.495 †	—	—	—
Cutaneous LP	2.826	16.87	4.20–67.80	2.636	13.96	3.94–55.84	<0.001
Genital LP	2.508	12.29	2.45–61.55	2.323	10.21	2.36–51.57	0.002
Autoimmune thyroiditis	2.718	15.14	3.47–66.11	2.539	12.67	3.26–54.88	<0.001
Oral Candida superinfection	1.587	4.89	1.11–21.52	1.499	4.48	1.10–18.82	0.037

ML, maximum likelihood; OR, odds ratio; CI, confidence interval; LR, likelihood ratio. † Intercept re-estimated with Firth’s intercept correction (FLIC), used for the calculation of predicted probabilities (Firth intercept before correction: −3.453). Predicted probability = 1/(1 + exp(−[−3.495 + 2.636 × cutaneous LP + 2.323 × genital LP + 2.539 × thyroiditis + 1.499 × Candida])).

**Table 4:** Performance of the final prediction model: apparent and optimism-corrected estimates (*n* = 141; 23 events).

Performance Measure	Apparent	Optimism-Corrected *	Ideal Value
AUC (95% CI)	0.889 (0.801–0.976)	0.840	1
Calibration intercept (calibration-in-the-large)	0.000	0.038	0
Calibration slope	1.073	0.733	1
Brier score	0.069	0.093	0
Scaled Brier score	0.496	0.319	1
Nagelkerke R^2^	0.539	0.357	1
Expected/observed events	23.0/23 (E/O = 1.00)	—	1

* Bootstrap internal validation (1000 samples), repeating backward selection and Firth penalized estimation in each sample; optimism summarized by the median across bootstrap samples. AUC 95% CI by the DeLong method. Scaled Brier score = 1 − Brier/Brier of a non-informative model (0.137). Calibration intercept and E/O are 0 and 1 by construction in the development data after intercept correction; the apparent calibration slope slightly above 1 reflects the shrinkage of coefficients by Firth penalization.

## Data Availability

The original contributions presented in this study are included in the article/supplementary material. Further inquiries can be directed to the corresponding author.

## Supplementary Materials

The following supporting information can be downloaded at: https://www.mdpi.com/article/10.3390/medicina62102041/s1, Supplementary Tables S1–S24: I. General characteristics of the study population: Table S1: Gender distribution of the study population, Table S2: Baseline demographic characteristics of the study cohort by age and gender, Table S3: Clinical phenotypes and baseline therapeutic regimens within the study cohort, Table S4: Intra-oral subsite distribution and extramucosal involvement in the studied OLP cohort, Table S5: Disease duration, globally and by genders, Table S6: Concurrent systemic therapies, supplementation, and dental material exposures in OLP patients, Table S7: Systemic comorbidities, autoimmune disorders, and localized infectious complications within the study cohort, Table S8: Treatment response, Table S9: Descriptive statistics of continuous clinical and laboratory variables; II. Comparative analysis between patients requiring systemic therapy and those managed without systemic therapy: univariate analysis for risk factor identification: Table S10: Association between gender and systemic therapy requirement in patients with oral lichen planus, Table S11: Univariate analysis of age according to systemic therapy status in patients with oral lichen planus, Table S12: Univariate analysis of clinical characteristics associated with the need for systemic therapy in patients with oral lichen planus, Table S13: Univariate analysis of oral lesion distribution according to systemic therapy status in patients with oral lichen planus, Table S14: Univariate analysis of extraoral disease involvement associated with the need for systemic therapy in patients with oral lichen planus, Table S15: Univariate analysis of disease duration according to systemic therapy status in patients with oral lichen planus, Table S16: Univariate analysis of disease duration according to systemic therapy status in patients with oral lichen planus, Table S17: Univariate analysis of comorbidities and clinical factors according to systemic therapy status in patients with oral lichen planus, Table S18: Univariate analysis of inflammatory biomarkers according to systemic therapy status in patients with oral lichen planus, Table S19: Univariate analysis of treatment response according to systemic therapy status in patients with oral lichen planus; III. Multivariate analysis for the identification of independent risk factors for systemic therapy in patients with oral lichen planus: Table S20: Observed and model-predicted probability of systemic therapy escalation for the 16 possible predictor profiles, Table S21: Bootstrap internal validation (1000 bootstrap samples; full model-development procedure repeated in each sample), Table S22: Stability of variable selection and LASSO sensitivity analysis, Table S23: Classification performance of the model at selected predicted-probability thresholds, Table S24: Patient characteristics, systemic therapy escalation and model performance according to recruiting centre, and centre-adjusted model.

## Abbreviations

The following abbreviations are used in this manuscript:

AAOMP	American Academy of Oral and Maxillofacial Pathology
AUC	Area Under the Curve
CBC	Complete Blood Count
CI	Confidence Interval
ESR	Erythrocyte Sedimentation Rate
HBV	Hepatitis B Virus
HCV	Hepatitis C Virus
HTN	Hypertension
IBD	Inflammatory Bowel Disease
IQR	Interquartile Range
LP	Lichen Planus
NLR	Neutrophil-to-Lymphocyte Ratio
OLP	Oral Lichen Planus
OR	Odds Ratio
ROC	Receiver Operating Characteristic
ROS	Reactive Oxygen Species
SD	Standard Deviation
T2DM	Type 2 Diabetes Mellitus
TNF-α	Tumor Necrosis Factor Alpha
IL	Interleukin
NF-κB	Nuclear Factor Kappa B
WHO	World Health Organization
