# Supplementary material for: Development of a Clinical Prediction Model for Systemic Therapy Escalation in Oral Lichen Planus: A Two-Center Retrospective Cohort Study
Source: Medicina (Kaunas). 2026 Oct 21;62(10):2041. doi: 10.3390/medicina62102041 (PMC13707202; doi:10.3390/medicina62102041)
Supplement: Supplementary file 1 [file medicina-62-02041-s001.zip › medicina-4508779-supplementary.pdf]

Supplementary materials

Manuscript title: Development of a Clinical Prediction Model for Systemic Therapy Escalation in Oral Lichen Planus: A Two-Center Retrospective Cohort Study

Authors: Mihaela Paula Toader, Cristina Popa, Cristina Colac-Boţoc, Ana Maria Sciucă, Anastasia Vicovan, Daciana Elena Brănişteanu

Contents:

I. General characteristics of the study population
II. Comparative analysis between patients requiring systemic therapy and those managed without systemic therapy: univariate analysis for risk factor identification
III. Multivariate analysis for the identification of independent risk factors for systemic therapy in patients with oral lichen planus

I. General characteristics of the study population

Table S1: Gender distribution of the study population

		n	%
Gender	Male	40	28.4
	Female	101	71.6
Total		141	100.0

Table S2: Baseline demographic characteristics of the study cohort by age and gender

a) Quantitative analysis:

Gender	n	Mean age	Standard deviation	Min	Max	Median	IQR	Mann-Whitney test
Male	40	65.00	13.089	21	88	67.00	58.50 ± 74.75	U = 1985.000
Female	101	65.53	13.307	22	95	67.00	57.00 ± 74.00	p = 0.873
Total	141	65.38	13.201	21	95	67.00	58.00 ± 74.00	

B) Qualitative analysis:

Age group	Male (n = 40)	Female (n = 101)	Total (n = 141)	Test	p	OR (95% CI)
<50 years	4 (10.0%)	8 (7.9%)	12 (8.5%)	Fisher–Freeman–Halton exact	0.940	-
50-64 years	12 (30.0%)	32 (31.7%)	44 (31.2%)			
65-74 years	14 (35.0%)	39 (38.6%)	53 (37.6%)			
≥75 years	10 (25.0%)	22 (21.8%)	32 (22.7%)			

Table S3: Clinical phenotypes and baseline therapeutic regimens within the study cohort

		n	%
Systemic treatment OLP	No	118	83.7
	Yes	23	16.3
Topical treatment OLP	No	39	27.7
	Yes	102	72.3

OLP- erosive subtype	No	80	56.7
	Yes	61	43.3
OLP- atrophic subtype	No	122	86.5
	Yes	19	13.5
OLP- reticular subtype	No	57	40.4
	Yes	84	59.6
OLP- plaque-like	No	121	85.8
	Yes	20	14.2
OLP- other subtypes	No	129	91.5
	Yes	12	8.5
Total		141	100.0

Note: Percentages for clinical forms do not sum to 100% due to the presence of overlapping/concurrent morphological patterns in individual patients

Table S4: Intra-oral subsite distribution and extramucosal involvement in the studied OLP cohort.

		n	%
Buccal mucosa	No	24	17.0
	Yes	117	83.0
Tongue	No	86	61.0
	Yes	55	39.0
Gingival tissue	No	125	88.7
	Yes	16	11.3
Hard palate	No	131	92.9
	Yes	10	7.1
Labial mucosa	No	123	87.2
	Yes	18	12.8
Cutaneous LP	No	120	85.1
	Yes	21	14.9
Genital LP	No	128	90.8
	Yes	13	9.2
Other sites	No	137	97.2
	Yes	4	2.8
Total		141	100.0

Table S5: Disease duration, globally and by genders:

A) Quantitative analysis:

Gender	n	Mean age	Standard deviation	Min	Max	Median	IQR	Mann-Whitney test
Male	40	7.30	5.548	0.00	21.00	6.00	2.00 ÷ 12.00	U = 1754.000
Female	101	6.47	6.569	0.00	42.00	4.00	1.00 ÷ 11.00	p = 0.223
Total	141	6.71	6.288	0.00	42.00	5.00	2.00 ÷ 11.25	

B) Qualitative analysis:

Disease	Male (n = 40)	Female (n =	Total	Test	p	OR (95% CI)

duration		101)	(n = 141)			
Recent (< 1 year)	3 (7.5%)	22 (21.8%)	25 (17.7%)	Pearson χ^2 = 4.271	0.118	-
Intermediate (1-3 years)	10 (25.0%)	18 (17.8%)	28 (19.9%)			
Long-standing (> 3 ani)	27 (67.5%)	61 (60.4%)	88 (62.4%)			

Table S6: Concurrent systemic therapies, supplementation, and dental material exposures in OLP patients.

		n	%
Systemic medication for associated conditions	No	54	38.3
	Yes	87	61.7
Mineral/ Vitamin supplementation	No	124	87.9
	Yes	17	12.1
Dental materials	No	46	32.6
	Yes	95	67.4
Total		141	100.0

Table S7.: Systemic comorbidities, autoimmune disorders, and localized infectious complications within the study cohort

		n	%
Hypertension	No	60	42.6
	Yes	81	57.4
Type 2 diabetes mellitus	No	108	76.6
	Yes	33	23.4
Dyslipidemia	No	114	80.9
	Yes	27	19.1
Chronic heart failure	No	129	91.5
	Yes	12	8.5
Obesity	No	121	85.8
	Yes	20	14.2
Inflammatory bowel disease	No	141	100.0
	Yes	-	-
Other autoimmune diseases (Sjogren syndrome, celiac disease, lupus, psoriasis, pernicious anemia)	No	129	91.5
	Yes	12	8.5
Thyroiditis	No	125	88.7
	Yes	16	11.3
Other thyroid disorders	No	125	88.7
	Yes	16	11.3
Psychiatric disorders	No	130	92.2
	Yes	11	7.8
Hepatitis B or C	No	118	83.7
	Yes	23	16.3
Anemia	No	130	92.2

	Yes	11	7.8
Candidosis	No	124	87.9
	Yes	17	12.1
Total		141	100.0

Table S8: Treatment response

		n	%
Treatment response	Absent	9	10.3
	Partial	14	16.1
	Complete	64	73.6
	Total (response documented)	87	100.0
	Response not documented	20	—
	No active treatment (not applicable)	34	—
Total		121	100.0

Table S9.: Descriptive statistics of continuous clinical and laboratory variables

	N	Mean	Standard deviation	Min	Max	Median	IQR
Disease duration (years)	141	6.71	6.288	0.00	42.00	5.00	2.00 ÷ 11.25
ESR	70	14.93	12.586	2.0	73.0	10.00	5.00 ÷ 20.00
NLR	117	2.18	1.333	0.17	9.03	1.83	1.40 ÷ 2.60

II. Comparative analysis between patients requiring systemic therapy and those managed without systemic therapy: univariate analysis for risk factor identification

Table S10: Association between gender and systemic therapy requirement in patients with oral lichen planus

**Variable**	**No systemic therapy (n = 118)**	**Systemic therapy (n = 23)**	**Test**	**p**	**OR (95% CI)**
Female sex	82 (69.5%)	19 (82.6%)	Pearson $\chi^2 = 1.630$	0.202	2.09 (0.66–6.57)

Table S11: Univariate analysis of age according to systemic therapy status in patients with oral lichen planus

OLP systemic treatment	n	Mean	Standard deviation	Min	Max	Median	IQR	Mann-Whitney test
No	118	66.25	13.069	21	95	68.00	60.00 ÷ 74.25	U = 980.000
Yes	23	60.96	13.272	22	92	61.00	53.00 ÷69.00	p = 0.035*

Table S12: Univariate analysis of clinical characteristics associated with the need for systemic therapy in patients with oral lichen planus

Variable	No systemic therapy (n = 118)	Systemic therapy (n = 23)	Test	p	OR (95% CI)
Topical treatment	84 (71.2%)	18 (78.3%)	Pearson $\chi^2 = 0.481$	0.488	1.46 (0.50–4.24)
Erosive	50 (42.4%)	11 (47.8%)	Pearson $\chi^2 = 0.233$	0.629	1.25 (0.51–3.05)
Atrophic	19 (16.1%)	0 (0.0%)	Fisher's exact	0.043	not estimable (0 events)
Reticular	68 (57.6%)	16 (69.6%)	Pearson $\chi^2 = 1.139$	0.286	1.68 (0.64–4.39)
Plaque-like	13 (11.0%)	7 (30.4%)	Fisher's exact	0.023	3.53 (1.23–10.19)
Other forms	11 (9.3%)	1 (4.3%)	Fisher's exact	0.690	0.44 (0.05–3.60)

Table S13: Univariate analysis of oral lesion distribution according to systemic therapy status in patients with oral lichen planus

Variable	No systemic therapy (n = 118)	Systemic therapy (n = 23)	Test	p	OR (95% CI)
Buccal mucosa	99 (83.9%)	18 (78.3%)	Fisher's exact	0.546	0.69 (0.23–2.09)
Tongue	48 (40.7%)	7 (30.4%)	Pearson $\chi^2 = 0.849$	0.357	0.64 (0.24–1.67)
Gingiva	12 (10.2%)	4 (17.4%)	Fisher's exact	0.298	1.86 (0.54–6.38)
Hard palate	6 (5.1%)	4 (17.4%)	Fisher's exact	0.058	3.93 (1.01–15.24)
Labial mucosa	14 (11.9%)	4 (17.4%)	Fisher's exact	0.496	1.56 (0.46–5.27)

Table S14: Univariate analysis of extraoral disease involvement associated with the need for systemic therapy in patients with oral lichen planus

Variable	No systemic therapy (n = 118)	Systemic therapy (n = 23)	Test	p	OR (95% CI)
Cutaneous LP	9 (7.6%)	12 (52.2%)	Fisher's exact	<0.001	13.21 (4.56–38.28)
Genital LP	4 (3.4%)	9 (39.1%)	Fisher's exact	<0.001	18.32 (4.98–67.36)
Other extraoral sites	2 (1.7%)	2 (8.7%)	Fisher's exact	0.125	5.52 (0.74–41.40)

Table S15: Univariate analysis of disease duration according to systemic therapy status in patients with oral lichen planus

Systemic treatment	n	Mean	Standard deviation	Min	Max	Median	IQR	Mann-Whitney test
No	118	6.98	5.531	0	21.00	6	2.00 ± 12.00	U = 912.500
Yes	23	5.31	9.301	0	42	1	0.25 ± 8.00	p=0.013*

Table S16: Univariate analysis of disease duration according to systemic therapy status in patients with oral lichen planus

Disease duration	No systemic therapy, n (%)	Systemic therapy, n (%)	Test
Recent (<1 year)	15 (12.7)	10 (43.5)	Pearson $\chi^2 = 12.747$ (df = 2)
Intermediate (1–3 years)	24 (20.3)	4 (17.4)	p = 0.002
Long-standing (>3 years)	79 (66.9)	9 (39.1)	Fisher–Freeman–Halton exact p = 0.003

Total	118 (100.0)	23 (100.0)	

Table S17: Univariate analysis of comorbidities and clinical factors according to systemic therapy status in patients with oral lichen planus

Variable	No systemic therapy (n = 118)	Systemic therapy (n = 23)	Test	p	OR (95% CI)
Systemic medication	74 (62.7%)	13 (56.5%)	Pearson χ^2 = 0.312	0.576	0.77 (0.31–1.91)
Mineral/ Vitamin supplements	16 (13.6%)	1 (4.3%)	Fisher's exact	0.307	0.29 (0.04–2.30)
Dental materials	86 (72.9%)	9 (39.1%)	Pearson χ^2 = 9.975	0.002	0.24 (0.09–0.61)
Hypertension	69 (58.5%)	12 (52.2%)	Pearson χ^2 = 0.313	0.576	0.77 (0.32–1.90)
Type 2 Diabetes Mellitus	27 (22.9%)	6 (26.1%)	Pearson χ^2 = 0.110	0.740	1.19 (0.43–3.32)
Dyslipidemia	18 (15.3%)	9 (39.1%)	Fisher's exact	0.017	3.57 (1.35–9.48)
Chronic heart failure	11 (9.3%)	1 (4.3%)	Fisher's exact	0.690	0.44 (0.05–3.60)
Obesity	13 (11.0%)	7 (30.4%)	Fisher's exact	0.023	3.53 (1.23–10.19)
Other autoimmune diseases (Sjogren syndrome, celiac disease, lupus, psoriasis, pernicious anemia)	8 (6.8%)	4 (17.4%)	Fisher's exact	0.108	2.89 (0.79–10.57)
Autoimmune thyroiditis	8 (6.8%)	8 (34.8%)	Fisher's exact	<0.001	7.33 (2.40–22.45)
Other thyroid disorders	13 (11.0%)	3 (13.0%)	Fisher's exact	0.726	1.21 (0.32–4.64)
Psychiatric disorders	8 (6.8%)	3 (13.0%)	Fisher's exact	0.388	2.06 (0.50–8.45)
HBV/HCV	21 (17.8%)	2 (8.7%)	Fisher's exact	0.368	0.44 (0.10–2.02)
Anemia	8 (6.8%)	3 (13.0%)	Fisher's exact	0.388	2.06 (0.50–8.45)
Oral Candida superinfection	11 (9.3%)	6 (26.1%)	Fisher's exact	0.035	3.43 (1.12–10.51)

Table S18: Univariate analysis of inflammatory biomarkers according to systemic therapy status in patients with oral lichen planus

Systemic treatment OLP	N	Mean	Standard deviation	Min	Max	Median	IQR	Mann-Whitney test
ESR - No	51	15.57	13.490	2.0	73.0	10.00	5.00 ÷ 20.00	U = 442.500
ESR - Yes	19	13.21	9.875	5.0	35.0	10.00	5.00 ÷ 15.00	p = 0.574
NLR - No	97	2.18	1.433	0.17	9.03	1.71	1.40 ÷ 2.60	U = 777.500
NLR - Yes	20	2.18	0.695	0.98	3.63	2.14	1.67 ÷ 2.62	p = 0.163

Table S19: Univariate analysis of treatment response according to systemic therapy status in patients with oral lichen planus

		Systemic treatment				Pearson Chi-square test
		No		Yes		
		n	%	n	%	
Treatment response	Absent	8	11.6	1	5.6	Chi2 =19.335
	Partial	5	7.2	9	50.0	p <0.001**
	Complete	56	81.2	8	44.4	
Total		69	100.0	18	100.0	

III. Multivariate analysis for the identification of independent risk factors for systemic therapy in patients with oral lichen planus

Table S20: Observed and model-predicted probability of systemic therapy escalation for the 16 possible predictor profiles.

Cutaneous LP	Genital LP	Thyroiditis	Candida	Patients (n)	Systemic therapy, observed n (%) [95% CI]	Firth penalized model - Predicted probability, % (95% CI)
−	−	−	−	92	3 (3.3%) [1.1–9.2]	2.9 (0.4–6.5)
−	−	−	+	10	0 (0.0%) [0.0–27.8]	12.0 (2.8–24.1)
−	−	+	−	9	2 (22.2%) [6.3–54.7]	27.8 (9.9–54.6)
−	+	−	−	3	1 (33.3%) [6.1–79.2]	23.7 (3.5–69.4) ‡
+	−	−	−	10	2 (20.0%) [5.7–51.0]	29.8 (11.3–55.1)
−	−	+	+	3	3 (100.0%) [43.8–100.0]	63.3 (18.8–91.1) ‡
−	+	−	+	1	1 (100.0%) [20.7–100.0]	58.1 (9.5–94.4) ‡
−	+	+	−	1	1 (100.0%) [20.7–100.0]	79.7 (31.9–98.5) ‡
+	−	−	+	2	2 (100.0%) [34.2–100.0]	65.5 (26.0–89.2) ‡
+	−	+	−	2	2 (100.0%) [34.2–100.0]	84.3 (58.4–97.4) ‡
+	+	−	−	7	6 (85.7%) [48.7–97.4]	81.2 (52.6–97.9)
−	+	+	+	1	0 (0.0%) [0.0–79.3]	94.6 (55.0–99.8) ‡
+	−	+	+	0	not observed	96.0 (73.5–99.7) *
+	+	−	+	0	not observed	95.1 (68.3–99.8) *
+	+	+	−	0	not observed	98.2 (89.7–99.9) *
+	+	+	+	0	not observed	99.6 (94.3–100.0) *

*+, present; −, absent. Observed proportions with 95% Wilson CIs. Predicted probabilities from the Firth penalized logistic regression model with intercept correction (FLIC); 95% CIs by non-parametric bootstrap (2,000 resamples, percentile method). * Profile not observed in the study cohort: model extrapolation; the CI reflects only coefficient uncertainty under the assumption of additive effects. ‡ Fewer than five patients.*

Table S21. Bootstrap internal validation (1,000 bootstrap samples; full model-development procedure repeated in each sample).

Procedure / performance measure	Apparent	Optimism	Optimism-corrected
A. Backward selection + ML estimation (unpenalized; for comparison)			
AUC	0.889	0.050	0.838
Calibration slope	1.000	0.307	0.693
Calibration-in-the-large	0.000	-0.045	0.045
Nagelkerke R^2	0.541	0.218	0.323
Brier score	0.068	-0.026	0.094
B. Backward selection + Firth/FLIC estimation (final model)			
AUC	0.889	0.048	0.840
Calibration slope	1.073	0.340	0.733
Calibration-in-the-large	0.000	-0.038	0.038
Nagelkerke R^2	0.539	0.182	0.357
Brier score	0.069	-0.024	0.093

C. Four prespecified predictors, Firth/FLIC (no selection)			
AUC	0.889	0.011	0.877
Calibration slope	1.073	0.116	0.957
Calibration-in-the-large	0.000	-0.036	0.036
Nagelkerke R^2	0.539	0.066	0.474
Brier score	0.069	-0.011	0.079

Optimism = median difference between performance in the bootstrap sample and in the original sample across 1,000 bootstrap samples; optimism-corrected = apparent − optimism. The median was used because a few bootstrap samples with quasi-complete separation yielded extreme calibration slopes (mean-based optimism-corrected slope for procedure A: 0.62). Apparent AUC 95% CI (DeLong): 0.801–0.976. Ideal values: calibration slope 1, calibration-in-the-large 0.

Table S22. Stability of variable selection and LASSO sensitivity analysis.

(a) Bootstrap inclusion frequencies of candidate predictors (backward selection, 1,000 bootstrap samples)

**Candidate predictor**	**Bootstrap inclusion frequency (%)**
Autoimmune thyroiditis	96.1
Cutaneous LP	92.0
Genital LP	85.2
Disease duration (years)	43.4
Oral Candida superinfection	43.2
Dental restorative materials	42.1
Dyslipidemia	41.1
Obesity	32.0
Age	21.4
Plaque-like phenotype	21.4
Recent-onset disease (<1 year)	17.9

(b) Most frequently selected models across bootstrap samples

**Selected model**	**Frequency among 1,000 bootstrap samples (%)**
Oral Candida superinfection + Cutaneous LP + Genital LP + Autoimmune thyroiditis	9.2
Oral Candida superinfection + Cutaneous LP + Genital LP + Obesity + Autoimmune thyroiditis	5.9
Dyslipidemia + Disease duration (years) + Cutaneous LP + Genital LP + Dental restorative materials + Autoimmune thyroiditis	4.3
Dyslipidemia + Cutaneous LP + Genital LP + Autoimmune thyroiditis	3.8
Cutaneous LP + Genital LP + Autoimmune thyroiditis	2.9

(c) LASSO-penalized logistic regression (all 11 candidate parameters; penalty chosen by 20 × 5-fold cross-validation)

**Candidate predictor**	**OR at λ(min)**	**OR at λ(1SE)**
Cutaneous LP	7.89	6.52
Autoimmune thyroiditis	7.05	5.50
Genital LP	5.99	5.23
Oral Candida superinfection	2.43	2.04
Dyslipidemia	1.76	1.55
Obesity	1.34	1.20
Dental restorative materials	0.72	0.83

Disease duration (per year)	1.00	not selected
Age; recent-onset disease; plaque-like phenotype	not selected	not selected

λ(min): penalty minimizing cross-validated deviance; λ(1SE): largest penalty within one standard error of the minimum. LASSO coefficients are shrunk towards zero by design and are reported to show the relative importance of predictors, not as unbiased effect estimates.

Table S23. Classification performance of the model at selected predicted-probability thresholds (apparent estimates, n = 141).

Threshold of predicted probability	Patients above threshold, n	Sensitivity	Specificity	PPV	NPV
≥10%	49	20/23 (87.0%)	89/118 (75.4%)	20/49 (40.8%)	89/92 (96.7%)
≥20%	39	20/23 (87.0%)	99/118 (83.9%)	20/39 (51.3%)	99/102 (97.1%)
≥50%	17	15/23 (65.2%)	116/118 (98.3%)	15/17 (88.2%)	116/124 (93.5%)

PPV, positive predictive value; NPV, negative predictive value. Estimates are apparent (not corrected for optimism) and are reported for descriptive purposes only; no threshold is proposed for clinical decision-making.

Table S24. Patient characteristics, systemic therapy escalation and model performance according to recruiting centre, and centre-adjusted model.

(a) Characteristics according to centre

Characteristic	Oral and Maxillofacial Surgery (n = 78)	Dermatology (n = 63)	p
Patients, n	78	63	—
Systemic therapy escalation	5 (6.4%)	18 (28.6%)	<0.001
Female sex	55 (70.5%)	46 (73.0%)	0.743
Cutaneous LP	6 (7.7%)	15 (23.8%)	0.008
Genital LP	3 (3.8%)	10 (15.9%)	0.014
Autoimmune thyroiditis	4 (5.1%)	12 (19.0%)	0.010
Oral Candida superinfection	6 (7.7%)	11 (17.5%)	0.077
Erosive phenotype	30 (38.5%)	31 (49.2%)	0.200
Atrophic phenotype	13 (16.7%)	6 (9.5%)	0.217
Reticular phenotype	45 (57.7%)	39 (61.9%)	0.612
Plaque-like phenotype	6 (7.7%)	14 (22.2%)	0.014
Dental restorative materials	63 (80.8%)	32 (50.8%)	<0.001
Dyslipidemia	13 (16.7%)	14 (22.2%)	0.405
Obesity	7 (9.0%)	13 (20.6%)	0.048
Hypertension	49 (62.8%)	32 (50.8%)	0.151
Type 2 diabetes mellitus	18 (23.1%)	15 (23.8%)	0.919
Age, years, median	70.0	65.0	0.002
Disease duration, years, median	8.5	1.0	<0.001

Values are n (%) unless otherwise specified. Pearson χ^2 when all expected frequencies ≥5, otherwise Fisher's exact test; Mann–Whitney U test for age and disease duration.

(b) Final model with and without adjustment for centre (Firth penalized logistic regression, n = 141, 23 events)

Predictor	OR (95% CI)	p
Main model (without centre)		
Cutaneous LP	13.96 (3.94–55.84)	<0.001
Genital LP	10.21 (2.36–51.57)	0.002
Autoimmune thyroiditis	12.67 (3.26–54.88)	<0.001

Oral Candida superinfection	4.48 (1.10–18.82)	0.037
Model adjusted for centre		
Cutaneous LP	11.82 (3.33–46.89)	<0.001
Genital LP	8.67 (2.02–43.15)	0.003
Autoimmune thyroiditis	10.26 (2.50–45.99)	0.001
Oral Candida superinfection	3.91 (0.94–16.57)	0.060
Dermatology centre (vs. OMF surgery)	2.05 (0.59–7.62)	0.257

Profile penalized-likelihood 95% CIs and penalized likelihood-ratio p-values. Centre-by-predictor interactions (each tested separately): all p > 0.35.

(c) Observed and model-expected events and discrimination in each centre (final model, not including centre)

**Centre**	**n**	**Observed events**	**Expected events (model)**	**AUC (95% CI)**
Oral and Maxillofacial Surgery	78	5 (6.4%)	6.9 (8.9%)	0.764 (0.481–1.000)
Dermatology	63	18 (28.6%)	16.1 (25.5%)	0.893 (0.801–0.984)

Expected events = sum of predicted probabilities. AUC 95% CI by the DeLong method (truncated at 1); centre-specific AUCs are imprecise because of the small number of events, particularly in the Oral and Maxillofacial Surgery centre (5 events).
